# Preliminary Results of Developing Imaging Complexity Biomarkers for the Incidence of Severe Radiation Pneumonitis Following Radiotherapy in Non-Small Cell Lung Cancer Patients with Underlying Idiopathic Pulmonary Fibrosis

**DOI:** 10.3390/life14070897

**Published:** 2024-07-19

**Authors:** Jeongeun Hwang, Hakyoung Kim, Sun Myung Kim, Dae Sik Yang

**Affiliations:** 1 Department of Medical IT Engineering, Soonchunhyang University, Asan 31538, Chungcheongnam-do, Republic of Korea; hwangje02@sch.ac.kr; 2 Departments of Radiation Oncology, Korea University Guro Hospital, Korea University College of Medicine, Seoul 08308, Republic of Korea; sunmyung01@hanmail.net

**Keywords:** non-small cell lung cancer, idiopathic pulmonary fibrosis, radiotherapy, complication, biomarker

## Abstract

**Background:** Idiopathic pulmonary fibrosis (IPF) has the potential to cause fatal pulmonary toxicity after radiotherapy and can increase the morbidity and mortality of non-small-cell lung cancer (NSCLC) patients. In this context, we aimed to develop imaging complexity biomarkers to predict the incidence of severe pulmonary toxicity in patients with NSCLC who have underlying IPF and are treated with radiotherapy. **Methods:** We retrospectively reviewed the medical records of 19 patients with NSCLC who had underlying IPF and were treated with radiotherapy at the Korea University Guro Hospital between March 2018 and December 2022. To quantify the morphometric complexity of the lung parenchyma, box-counting fractal dimensions and lacunarity analyses were performed on pre-radiotherapy simulation chest computed tomography scans. **Results:** Of the 19 patients, the incidence of grade 3 or higher radiation pneumonitis was observed in 8 (42.1%). After adjusting for age, sex, smoking status, histology, and diffusing capacity of the lung for carbon monoxide, eight patients with a lower fractal dimension showed a significantly higher hazard ratio of 7.755 (1.168–51.51) for grade 3 or higher pneumonitis than those with a higher fractal dimension. Patients with lower lacunarity exhibited significantly lower hazards in all models, both with and without adjustments. The lower-than-median lacunarity group also showed significantly lower incidence curves for all models built in this study. **Conclusions:** We devised a technique for quantifying morphometric complexity in NSCLC patients with IPF on radiotherapy and discovered lacunarity as a potential imaging biomarker for grade 3 or higher pneumonitis.

## 1. Introduction

Idiopathic pulmonary fibrosis (IPF) is characterized as a lung disease of unknown cause that worsens progressively, resulting in mortality [1,2]. Considering the prognosis, patients with IPF have a median survival rate of 2.5–3.5 years. Moreover, the 5-year survival rate of patients with IPF ranges from 20 to 40%. Irreversible and chronic progression are typical characteristics of IPF. The clinical course of IPF can vary from a slow progressive course for many months to years to a rapid, progressive course accompanied by an abrupt exacerbation of pulmonary symptoms and deteriorating lung function.

In particular, for patients with non-small-cell lung cancer (NSCLC) who have underlying IPF [3,4,5,6,7,8], cancer treatment, which includes radiotherapy, surgery, and chemotherapy, can result in substantial treatment-related complications. Among these, IPF has the potential to cause fatal pulmonary toxicity after radiotherapy and increase the morbidity and mortality of NSCLC patients [9,10,11,12].

Currently, no drugs are available for treating IPF [13]. Only two antifibrotic drugs, pirfenidone and nintedanib, have been approved. However, these drugs only slow the rate of fibrosis or scarring in the lungs and play a limited role in preventing rapid exacerbations of the disease [14,15,16]. Recently, efforts have been undertaken to actively administer antifibrotic and anti-inflammatory drugs, such as Pirfenidone (Pirespa^®^), to patients diagnosed with IPF.

In this context, we aimed to develop imaging complexity biomarkers to predict the incidence of severe pulmonary toxicity in patients with NSCLC who have underlying IPF and are treated with radiotherapy. Patients with chronic obstructive pulmonary disease (COPD) presenting with lower morphometric complexity in their lung parenchyma had poor prognoses [17]. However, whether the association found in patients with COPD also applies to NSCLC patients with IPF undergoing radiotherapy is yet to be determined. In a previous study [17], box-counting fractal dimension measurements represented a morphological aspect of the parenchymal integrity of the lung. Assuming an analogy to NSCLC patients with IPF, a lower risk of severe radiation pneumonitis may be expected in patients with higher parenchymal integrity in the lung, who would have higher morphometric complexity. We aimed to develop a methodology for measuring morphometric complexity in NSCLC patients with IPF undergoing radiotherapy and to establish a framework to utilize complexity measurements for the prognosis of severe radiation pneumonitis.

## 2. Materials and Methods

### 2.1. Patients

We retrospectively reviewed the medical records of 257 patients with NSCLC who underwent thoracic X-ray radiotherapy at the Korea University Guro Hospital between March 2018 and December 2022. All diagnoses of the underlying pulmonary diseases were confirmed by experienced pulmonologists (J.H.C.). Among them, patients with no underlying pulmonary disease other than IPF, such as COPD, and those lacking pulmonary function tests were excluded; 19 patients with underlying IPF were included in this study.

### 2.2. Diagnostic Scheme for Lung Cancer and IPF

Tumor assessment included documenting a complete medical history, physical examination, complete blood counts, chemistry profiles, pulmonary function test (PFT), chest radiography, computed tomography (CT) of the chest and upper abdomen, whole-body 18F-fluorodeoxyglucose positron emission tomography with CT (FDG-PET-CT), and magnetic resonance imaging of the brain as a routine staging work-up. PFT, including both spirometry and diffusion capacity, was performed before treatment. Detailed measurements included the following: (1) forced expiratory volume in 1 s (FEV1), (2) forced vital capacity (FVC), (3) ratio of the two volumes (FEV1/FVC), and (4) diffusing capacity of the lung for carbon monoxide (DLCO). The diagnosis of IPF is made based on the presence of a typical radiological pattern, which is a coarse reticulation with a honeycombing appearance in the peripheral and predominantly basal lung areas on high-resolution CT. Spirometry typically reveals a reduction in vital capacity and DLCO. The GAP model, which includes four baseline variables (sex, age, and two lung physiology variables, FVC and DLCO), was used for IPF staging [18].

### 2.3. Treatment Scheme and Surveillance

Based on the institutional protocol, stereotactic ablative radiation therapy (SABR) with a total dose of 60 Gy in four fractions was administered to NSCLC patients with small-sized (≤4 cm) and peripherally located tumors. For patients who received intensity-modulated radiation therapy (IMRT), two different dose-fractionation schedules were planned to deliver 60 Gy in 20 fractions in the radiotherapy-alone group and 66/60 Gy in 30 fractions in the concurrent chemoradiotherapy group using the simultaneous integrated boost technique. 

For target delineation of lung and mediastinal nodal lesions in IMRT planning, the gross tumor volume (GTV) was delineated under the lung and mediastinal window settings. The internal target volume (ITV) was delineated following four-dimensional CT with special regard to the patient’s respiratory motion. The clinical target volume (CTV) was generated with a 5 mm expansion of the GTV-ITV in all directions and then modified according to the adjacent normal anatomic structures. The planning target volume (PTV) was generated with a 5 mm expansion of the CTV. The prescription guideline was to deliver at least 97% of the prescribed dose to 95% of the PTV. The minimum and maximum doses to 1cc of PTV were 95% and 107%, respectively.

For target delineation of lung lesions in SBRT planning, the GTV was delineated under the lung window settings. The GTV-ITV was delineated following four-dimensional CT with special regard to the patient’s respiratory motion. The PTV was generated with a 5 mm expansion of the GTV-ITV. The prescription guideline was that 95% of PTV should be covered by prescription dose. The percentage lung volume that received ≥20 Gy was to be kept ≤35%, and the mean lung dose was ≤20 Gy. The maximum doses to the spinal cord and esophagus were not to exceed 45 Gy and 60 Gy, respectively, satisfying the dose-volume constraints of normal organs.

Physical examination, blood tests, chest CT, and/or PET-CT were performed every 3 months for 2 years after radiotherapy and then every 6 months thereafter to detect disease progression during follow-up. Treatment-related complications were evaluated using the Common Terminology Criteria for Adverse Events (version 4.03).

### 2.4. Morphometric Complexity Measurements

**Figure 1:**
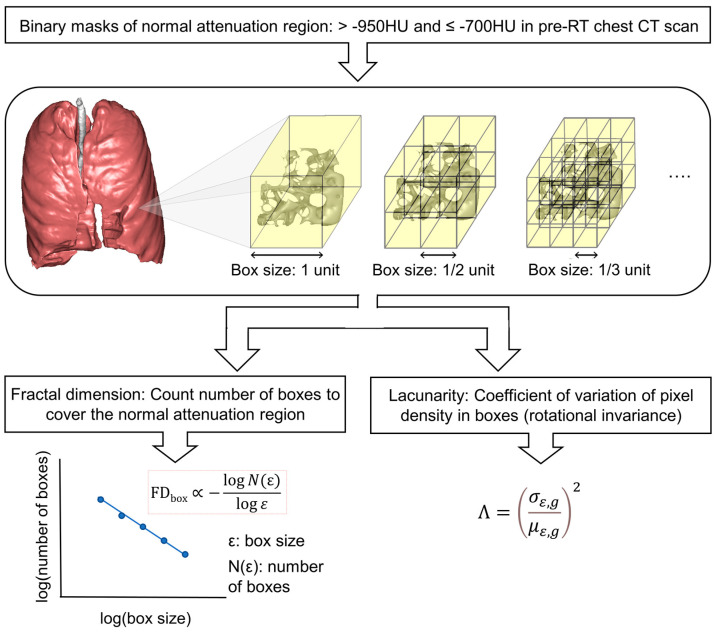
Process of morphometric complexity analysis on pre-radiotherapy chest CT scans. The normal attenuation region masks were binarized at >−950 HU and ≤−700 HU. A box counting method was applied at the binary masks. In fractal dimension analysis, number of boxes *N*(ε) needed to cover the mask was counted at varying box-sizes ε. In lacunarity measurement, a probability density of pixels belonging to the mask were counted within an arbitrary box size epsilon and orientation g, and the lacunarity Λ was calculated by the square of the standard deviation *σ_ε,g_* and mean *μ_ε,g_*.

To quantify the morphometric complexity of the lung parenchyma, box-counting fractal dimensions and lacunarity analyses were performed on a pre-radiotherapy-simulated chest CT scan. The model name is Canon’s Aquilion Lightning 80 (KV-CT, DLP (mGy) effective dose). In radiation treatment planning, IMRT planning used 6 MV energy, and SBRT planning used 6 FFF energy. Figure 1 shows the process and the morphometric analysis schema used in this study. Binary masks of intact lung parenchyma were defined as normal attenuation regions at >−950 HU and ≤−700 HU. An in-house box-counting fractal analysis tool was built according to the algorithm suggested by Grassberger [19] and Ott et al. [20] using MATLAB release 2022a (MathWorks Inc., Natick, MA, USA), with a higher dimension implying greater spatial filling. In brief, the number of imaginary cubes with sizes of 2, 4, 8, 16, and 32 voxel lengths needed to cover the normal attenuation region mask was counted, and a logarithmic regression was performed to estimate the power-law exponent, as in Formula (1):

(1) $${FD}_{box}\left(M\right)\propto \frac{\log N(\varepsilon )}{\log\left(\frac{1}{\varepsilon }\right)}$$

where *M* is the 3D mask of the normal attenuation region, *ε* is the size of cubes, and *N*(*ε*) is the number of size *ε* cubes needed to cover the mask.

Lacunarity is another measure of spatial heterogeneity but differs from the fractal dimension in that it measures rotation invariance [21]. Lacunarity analysis also utilizes a box-counting method, in which the probability density of pixels belonging to the mask is counted within each box size. Formula (2) shows how a lacunarity Λ is calculated:

(2) $$\Lambda ={\left(\frac{\sigma_{\varepsilon ,g}}{\mu_{\varepsilon ,g}}\right)}^{2}$$

where *σ_ε,g_* stands for the standard deviation of box-size *ε* and orientation *g*, *μ_ε,g_* is for the average [21]. A higher lacunarity measurement denotes higher rotational variance and a more heterogeneous spatial distribution of the normal attenuation region mask [21].

### 2.5. Statistical Analyses

The data are reported as numbers for categorical variables and medians for continuous variables. Time-to-event data on the occurrence of grade ≥ 3 radiation pneumonitis were used. Cox proportional hazard models [22] were built to assess the hazards of morphometric complexity measures for radiation pneumonitis. Hazard ratios, 95% confidence intervals, and Harrell’s C-indices were calculated with or without adjustment for age, sex, smoking status, histology, and pre-radiotherapy DLCO. Proportional hazard assumptions were investigated by performing chi-square tests for the Shoenfeld residuals. Statistical significance was set at *p* < 0.05. All statistical analyses were performed using R statistics software version 4.3.1 (R Foundation for Statistical Computing, Vienna, Austria).

## 3. Results

### 3.1. Clinical Characteristics

Table 1 summarizes the clinical characteristics of the patients. The median age of the study population was 74 years (range, 53–86 years). Most patients were men (94.7%) and current or ex-smokers (89.5%). Prior to the initiation of radiation therapy, 50% of the patients with IPF were using anti-fibrotic medications. Most patients showed impaired pulmonary function with a DLCO of less than 60% (73.7%). Regarding the radiotherapy technique, 13 (68.4%) and 6 (31.6%) patients were treated with IMRT and SABR, respectively.

### 3.2. Treatment-Related Complications

The incidence of grade 3 or higher radiation pneumonitis was 42.1% (8/19) in patients with IPF, including one case of grade 5 radiation pneumonitis. In the remaining patient group excluding IPF, 17 out of 238 patients (7.1%) experienced grade 3 or higher radiation pneumonitis. Grade 5 complications were reported in a male patient who is a current smoker with underlying chronic kidney disease. In addition, the patient showed impaired pulmonary function (pre-radiotherapy DLCO: 40%), and no anti-fibrotic medication was taken. The patient was treated with definitive radiotherapy alone at a total dose of 60 Gy in 20 fractions using the IMRT technique. Severe radiation pneumonitis occurred 4 months after the completion of IMRT. The results of pulmonary function tests performed at that time were worse (post-radiotherapy DLCO, 31%). After hospitalization, the patient died of severe radiation pneumonitis and an exacerbation of the underlying disease, IPF, despite intensive treatment with steroids and mechanical ventilation.

### 3.3. Morphometric Complexity Analysis

**Figure 2:**
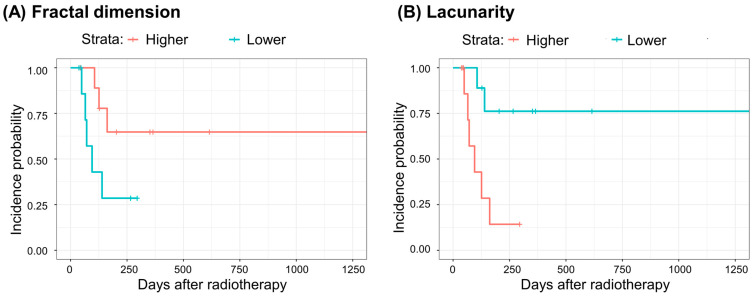
Grade ≥ 3 pneumonitis incidence curves according to (A) fractal dimension and (**B**) lacunarity strata. In both (**A**) and (**B**), a total of 19 patients were stratified to ‘Higher (red curves)’ and ‘Lower (blue curves)’ strata at median. (**A**) Higher fractal dimension, red curve: 10 patients with fractal dimension 2.175–2.363; lower fractal dimension, blue curve: 9 patients with fractal dimension 1.914–2.157, *p* = 0.070 in a log-rank test. (**B**) Higher lacunarity, red curve, 10 patients with lacunarity 0.455–0.908; lower lacunarity, blue curve, 9 patients with lacunarity 0.317–0.454; *p* = 0.007 in a log-rank test.

Box-counting fractal dimensions and lacunarity analyses were performed on all patients’ pre-radiotherapy chest CT scans. All patients were dichotomized at median values. The hazards of lower-than-median fractal dimension or lacunarity for grade ≥ 3 events were estimated by building Cox proportional hazard models with and without adjustment. The adjusted models included age, sex, smoking status, histology, and DLCO. Chi-square tests for the Schoenfeld residuals of each model showed no significant violation of the proportional hazards assumption. In addition, log-rank tests were performed on the incidence curves of the lower- and higher-than-median groups. Table 2 shows the hazard ratios and their 95% confidence intervals, *p*-values, Harrell’s C-indices, and *p*-values of the log-rank tests. After adjusting for age, sex, smoking status, histology, and DLCO, eight patients with a lower fractal dimension showed a significantly higher hazard ratio of 7.755 (1.168–51.51) for grade ≥ 3 pneumonitis than those with a higher fractal dimension. Patients with lower lacunarity exhibited significantly lower hazards in all models, both with and without adjustments. The lower-than-median lacunarity group also showed significantly lower incidence curves (Figure 2) for all models built in this study. 

We performed two alternative analyses sharing the same features and architects of models as previously described, except (1) by using grade ≥ 2 pneumonitis instead of grade ≥ 3 as outcome event definition or (2) by adjusting for GAP stage instead of age, sex, smoking status, histology, and DLCO in the multivariate regression model. All the alternative analyses qualitatively exhibited the same results as those of the previously described models.

## 4. Discussion

We attempted to identify CT morphometric complexity biomarkers representing normal lung tissue integrity in NSCLC patients who have underlying IPF, thereby associating them with the risk of severe radiation pneumonitis after radiotherapy. We adopted box-counting fractal dimension and lacunarity analyses. Both the fractal dimension and lacunarity are measures of morphometric complexity based on the box-counting method. The box-counting fractal dimension is a measure of the space-filling property, whereas lacunarity is a measure of rotation invariance. Fractal analysis has previously been used as a measure of lung parenchymal integrity in patients with COPD and is associated with survival [17]. In that study, the fractal analysis was on binary masks of the normal attenuation area, which was voxels ≥−950 HU within the lung [17]. The fractal dimension is more closely associated with COPD symptoms and survival [17,23] than the volume fraction of the low attenuation region, owing to the lung inflation-level robustness of the box-counting method. Because our study included NSCLC patients with underlying IPF, we modified the identification of normal attenuation areas as >−950 HU and ≤−700 HU within the lung. 

In fractal dimension analysis, we found a marginal association between a fractal dimension and grade ≥ 3 pneumonitis and a significant association when adjusted for age, sex, smoking status, histology, and DLCO in a way that patients with higher fractal dimension values had less hazard than those with lower values. We observed significant associations between a lower lacunarity value and less grade ≥ 3 pneumonitis hazard in both crude and adjusted multivariate Cox-proportional hazard models (Table 2 and Figure 2). We speculate that these morphometric characteristics indicate better parenchymal integrity in the lungs. Intuitive interpretation of a box counting fractal dimension is a space-filling property, and that of lacunarity is a ‘gappiness,’ or rotation invariance [21]. Airways and blood vessels in the lung are both tree structures, and they are expected to have high space-filling properties in a ‘healthy’ lung [24,25,26,27]. The inherent tree structures of the airways and blood vessels in the lungs may have high lacunarity values because of the high rotation variance and gappiness of the tree structures. Alternatively, normal lung parenchyma excluding emphysematous regions (≤−950 HU) and fibrotic, consolidated regions (>−700 HU) with good integrity would also have high space-filling properties but would have less gaps and have rotationally homogeneous structures. The association was significant for lacunarity and marginal for the fractal dimension; the reason for this discrepancy is unclear. This may imply that lacunarity is a more appropriate imaging biomarker for prognosis in NSCLC patients with underlying IPF receiving radiotherapy; however, the relatively small sample size of 19 patients precludes this conclusion.

We developed a methodology for measuring morphometric complexity in NSCLC patients with IPF undergoing radiotherapy and established a framework to utilize box-counting fractal dimension and lacunarity measurements for the prognosis of severe radiation pneumonitis, showing significant associations between lower lacunarity and less hazard. The present study showed associations, so further investigations with more participants and different study designs are needed to understand the detailed mechanism of this association between morphometric complexity and radiation pneumonitis hazards or to identify causal inferences. IPF itself is a rare disease; based on the results of the analysis of 19 patients in this study, we plan to expand future research to all interstitial lung disease and COPD disease groups.

## 5. Conclusions

We have devised a technique for quantifying morphometric complexity in NSCLC patients with IPF who are receiving radiotherapy and discovered lacunarity as a potential imaging biomarker for grade ≥ 3 pneumonitis.

## Figures and Tables

**Table 1:** Clinical characteristics (*N* = 19).

Characteristics	Number	%
Age [years; median (range)]	74 (53–86)	
Sex		
Female	1	5.3%
Male	18	94.7%
Smoking Status		
Never smoker	2	10.5%
Current or Ex-smoker	17	89.5%
Histology		
Adenocarcinoma	8	42.1%
Squamous cell carcinoma	11	57.9%
Anti-fibrotic agent treatment		
No	9	47.4%
Yes	10	52.6%
Pretreatment DLCO		
>60%	5	26.3%
≤60%	10	52.6%
≤40%	4	21.1%
Radiotherapy aim		
Definitive radiotherapy alone	6	31.6%
Definitive CCRT	6	31.6%
Consolidative radiotherapy alone	7	36.8%
Radiotherapy technique		
IMRT	13	68.4%
SABR	6	31.6%
GAP IPF stage		
I	7	36.8%
II	11	57.9%
III	1	5.3%

DLCO, diffusing capacity of the lung for carbon monoxide; CCRT, concurrent chemo-radiation therapy; IMRT, intensity modulated radiation therapy; SABR, stereotactic ablative radiation therapy.

**Table 2:** Associations between risk factors and incidence of radiation pneumonitis ≥ grade 3.

			Fractal Dimension	Lacunarity		
			2.175–2.363 (*N* = 10)	1.914–2.157 (*N* = 9)	0.455–0.908 (*N* = 10)	0.317–0.454 (N = 9)
**Grade** **≥** **3** **(*N* = 8)**	Unadjusted model	HR ^b^ (95% CI ^c^)	Reference	3.536 (0.833–15.01)	Reference	0.144 (0.029–0.725)
		*p*		0.087		0.019
		C-index ^d^		0.689		0.750
		p of Log-rank test		0.070		0.007
	Adjusted model ^a^	HR (95% CI)	Reference	7.755 (1.168–51.51)	Reference	0.022 (0.002–0.306)
		*p*		0.034		0.004
		C-index		0.744		0.878
		p of Log-rank test		0.200		0.020

^a^ Adjusted model: adjusted for age, gender, smoking status, histology, and DLCO%; ^b^ HR: hazard ratio; ^c^ CI: confidence interval; ^d^ C-index: Harrel’s C. Note: both fractal dimension and lacunarity are real numbers without any unit.

## Data Availability

The datasets used and/or analyzed in the current study can be obtained from the corresponding author upon reasonable request.
